# In Silico Design and Selection of New Tetrahydroisoquinoline-Based CD44 Antagonist Candidates

**DOI:** 10.3390/molecules26071877

**Published:** 2021-03-26

**Authors:** Angel J. Ruiz-Moreno, Atilio Reyes-Romero, Alexander Dömling, Marco A. Velasco-Velázquez

**Affiliations:** 1 Departamento de Farmacología, Facultad de Medicina, Universidad Nacional Autónoma de Mexico (UNAM), Ciudad de Mexico 04510, Mexico; angel_ruiz_m@comunidad.unam.mx; 2 Unidad Periférica de Investigación en Biomedicina Translacional, Facultad de Medicina, Universidad Nacional Autónoma de México (UNAM), Félix Cuevas 540, Ciudad de Mexico 03229, Mexico; 3 Doctorado en Ciencias Biomédicas, Universidad Nacional Autónoma de México (UNAM), Ciudad de Mexico 04510, Mexico; 4 Drug Design Group, Department of Pharmacy, University of Groningen, 9700 AD Groningen, The Netherlands; atilioreyesromero@gmail.com

**Keywords:** tetrahydroisoquinoline, CD44, computational combinatorial chemistry, pharmacophore, molecular dynamics

## Abstract

CD44 promotes metastasis, chemoresistance, and stemness in different types of cancer and is a target for the development of new anti-cancer therapies. All CD44 isoforms share a common N-terminal domain that binds to hyaluronic acid (HA). Herein, we used a computational approach to design new potential CD44 antagonists and evaluate their target-binding ability. By analyzing 30 crystal structures of the HA-binding domain (CD44HAbd), we characterized a subdomain that binds to 1,2,3,4-tetrahydroisoquinoline (THQ)-containing compounds and is adjacent to residues essential for HA interaction. By computational combinatorial chemistry (CCC), we designed 168,190 molecules and compared their conformers to a pharmacophore containing the key features of the crystallographic THQ binding mode. Approximately 0.01% of the compounds matched the pharmacophore and were analyzed by computational docking and molecular dynamics (MD). We identified two compounds, Can125 and Can159, that bound to human CD44HAbd (hCD44HAbd) in explicit-solvent MD simulations and therefore may elicit CD44 blockage. These compounds can be easily synthesized by multicomponent reactions for activity testing and their binding mode, reported here, could be helpful in the design of more potent CD44 antagonists.

## 1. Introduction

CD44 is a transmembrane glycoprotein that functions as a receptor for the glycosaminoglycan hyaluronic acid (HA), an integral component of the extracellular matrix [1,2]. CD44 is expressed on multiple cells, including embryonic stem cells and differentiated cells, mediating cellular functions such as adhesion, homing, migration, and extravasation [1,2]. CD44 transcript can undergo alternative splicing, generating multiple isoforms of CD44, but all of them conserve intact the HA-binding domain (HAbd) and, therefore, can be activated by HA [3].

CD44 expression correlates with unfavorable clinical outcomes in multiple types of cancer [4,5,6,7,8]. CD44 activation by HA in cancer cells induces transcriptional and epigenetic changes that stimulate signaling pathways controlling invasiveness and metastasis, chemoresistance, and stemness [9,10,11]. For instance, in breast cancer cells, HA binding to CD44 induces epithelial–mesenchymal transition, which increases cell migration and invasive capacity [12], and promotes survival under detached conditions during the development of metastasis [13]. Moreover, CD44 is expressed in cancer stem cells that survive chemotherapy in models of glioblastoma [14], breast [15], pancreatic [16], colorectal [17], and prostate [18] cancer. Consistent with its key role in cancer progression, CD44 silencing impairs chemoresistance, clonogenicity, tumorigenicity, and/or metastasis [19,20,21]. Therefore, blockage of HA-binding to CD44 has been proposed as a potential therapeutic strategy for cancer.

The CD44HAbd is located in the N-terminal end of the extracellular region of the receptor. Structural analysis of murine CD44HAbd crystals showed that only 13 residues along a shallow groove mediate HA-binding [22]. The residues Arg41, Tyr42, Arg78, Tyr79 in hCD44HAbd (Arg45, Tyr46, Arg82, Tyr83 in mCD44HAbd) have been previously described as essential for HA-binding by directed mutagenesis experiments or crystal analysis [23,24]. Given the lack of an obvious druggable pocket in the HA-binding site, small molecule inhibitors that interact with allosteric sites within the CD44HAbd have been developed [24,25,26,27]. However, those compounds bind to CD44HAbd in the high micromolar or even low millimolar range, limiting further applications. Therefore, there is a need for new CD44 antagonists with improved affinity, efficacy, and physicochemical properties for future effective translation to the clinic.

Herein we designed and evaluated the binding of new potential CD44 antagonists using an in silico strategy. We identified that small molecules sharing a 1,2,3,4-tetrahydroisoquinoline (THQ) motif are frequently co-crystallized with CD44HAbd in a subdomain adjacent to the residues that are essential for HA-binding. By computational combinatorial chemistry (CCC), we generated libraries including more than 168,000 THQ-containing molecules. The new molecules (i) could be easily synthesized by multicomponent reactions, (ii) are diverse, and (iii) display drug-like physicochemical properties. We selected a subset of 163 candidates matching the key features of the reported THQ binding mode for further analysis by computational docking. The nine candidates with the highest frequency of poses reproducing the reported THQ binding mode were analyzed by molecular dynamics (MD). Our results allowed the identification of two compounds predicted to stably bind to hCD44HAbd in an aqueous solution. Those compounds may be useful as CD44 antagonists, and the information of their binding mode can be employed as the basis for the design of new bioactive molecules that target CD44.

## 2. Results

### 2.1. Identification of a Target Subdomain within the CD44HAbd and Generation of a THQ-Based Pharmacophore

Aiming to identify relevant regions for drug design, we compared the 30 crystal structures available in Protein Data Bank (PDB) that comprise the HA-binding domain of human (three structures) or mouse (27 structures) CD44 (Table S1). The three human structures correspond to the apo form of CD44HAbd. For mCD44HAbd, 2JCP represents the apo-CD44HAbd, three structures are co-crystallized with HA (2JCQ, 2JCR, and 4MRD), and the rest are co-crystallized with molecules weighting 100–250 Da. Within the structures containing small molecules, 21 of them are co-crystallized with compounds containing the THQ motif. Sequence identity analysis showed 100% identity among all hCD44HAbd, 99–100% among mCD44HAbd, and 86–88% between hCD44HAbd and mCD44HAbd (Figure S1A). Due to the high identity among CD44HAbd structures, we compared all of them in a structural analysis. The root mean square deviation (RMSD) profiles for alpha-carbons and full atoms showed the higher deviations on some residues previously reported as essential for HA-binding by direct mutagenesis experiments (Arg41, Tyr42, Arg78, and Tyr79) in hCD44HAbd [23] (Figure S1B).

**Figure 1:**
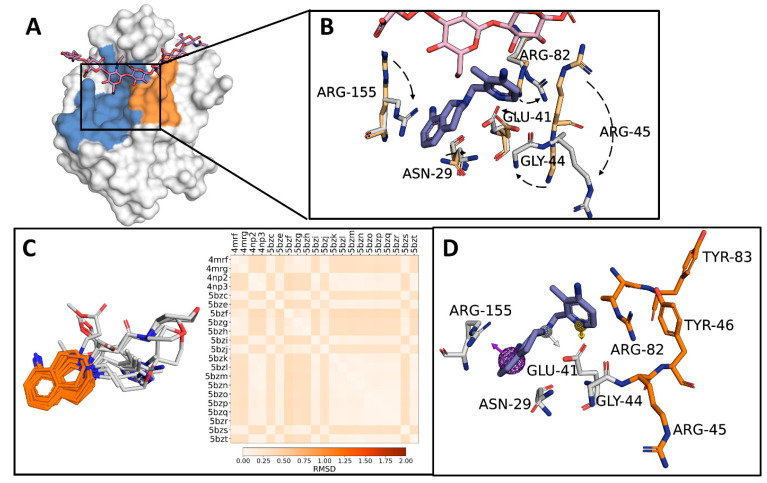
Generation of a 1,2,3,4-tetrahydroisoquinoline (THQ)-based pharmacophore. (**A**) CD44HAbd (white surface) has a subdomain where the THQ-containing molecules can bind (blue) and partially overlaps with the region containing the key residues for CD44–HA binding (Arg45, Tyr46, Arg82, and Tyr83-orange-). HA is shown in pink. (**B**) Spatial positions of the lateral chain of residues Asn29, Glu41, Gly44, Arg45, Arg82, and Arg155 from mCD44HAbd in the HA-bound form (residues shown as beige sticks; HA in pink) vs. the form bound to a THQ-containing molecule (residues shown as grey sticks; ligand in blue). Dashes represent the residue shifts between both states. (**C**) Structural alignment and root mean square deviation (RMSD) comparison of the THQ scaffold (orange) of the molecules co-crystallized with mCD44HAbd. (**D**) Pharmacophore model generated using THQ-containing molecules as a template. Purple sphere: aromatic; yellow sphere: hydrogen bond donor; white sphere: merged hydrogen bond donor and positively charged ion. A molecule with the THQ substructure is included (blue) to show the interacting residues in CD44 (grey sticks) and its proximity to residues essential for HA-binding (orange sticks).

Analysis of the 21 structures co-crystallized with THQ-containing small molecules identified that all these ligands bind to a pocket contiguous to the HA-binding domain (Figure 1A). Their binding drives a shift in multiple residues of mCD44HAbd, including some of the key residues participating in HA-binding, namely, Arg45, Arg82, and Arg155 (Figure 1B). Alignment of THQ-composing atoms showed that the binding mode is highly conserved among the 21 crystals analyzed (Figure 1C). Thus, we model a pharmacophore from the co-crystallized molecules containing the THQ substructure. The generated model included four pharmacophoric descriptors—aromatic, two hydrogen bond donors, and a positively charged ion (Figure 1D).

### 2.2. Generation of THQ-Containing Libraries

**Scheme 1:**
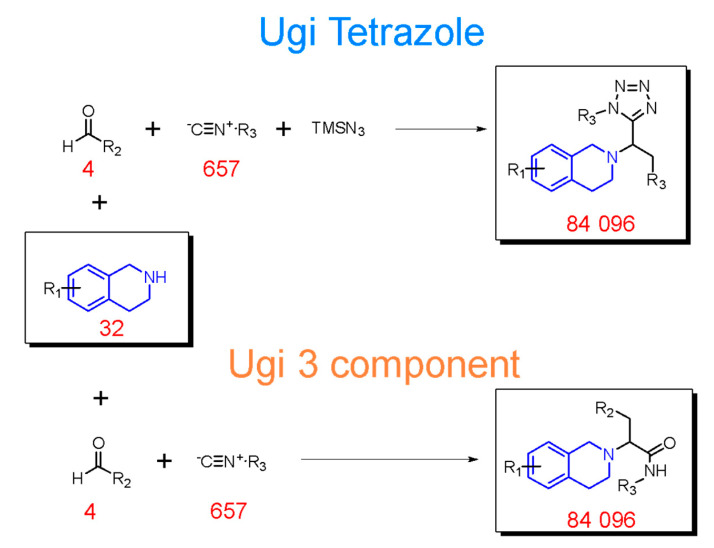
CCC strategy for the generation of the test libraries.

To identify new compounds with the potential capability to interfere with the CD44–HA binding, we employed CCC to generate two libraries of compounds that include the THQ scaffold. To facilitate the synthesis of our compounds in subsequent research, we decided to use multicomponent reactions (MCR) synthesis routes. Considering the characteristics of the THQ-containing small molecules co-crystallized with mCD44HAbd, we implemented the Ugi four-component tetrazole synthesis [28,29] and Ugi three-component reaction [30] for our CCC experiments. For each MCR route, we obtained 84,096 different compounds (Scheme 1).

### 2.3. Cheminformatic Analysis of CCC Compounds

**Figure 2:**
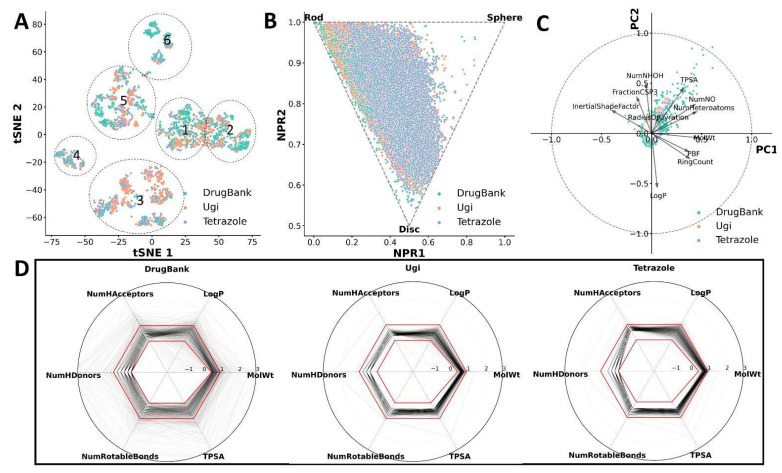
Characteristics of the generated libraries. (**A**) t-distributed stochastic neighbor embedding (tSNE) chart of structural diversity analysis for the compounds generated by Ugi three-component reaction (Ugi) or Ugi four-component tetrazole synthesis (Tetrazole). For comparison, a database containing FDA-approved molecules was included (DrugBank). (**B**) Normalized principal moments ratio (NPR) analysis. (**C**) Principal components analysis (PCA) for molecular and physicochemical descriptors. (**D**) Lipinski’s rule of five (Ro5) analysis, which included molecular weight (MolWt), logarithmic partition coefficient (LogP), number of hydrogen bond donors (NumHDonors) and acceptors (NumHAcceptors), and the topological polar surface area (TPSA). A gray line represents each compound, and the density indicates the frequency of compounds.

In order to explore the chemical diversity and physicochemical properties of the designed compounds, we employed a series of cheminformatic analyses, calculating 30 different 2D/3D-shape and physicochemical molecular descriptors. For comparison, we also studied the compounds within DrugBank Database 5.0.10, a library of 1542 FDA-approved small molecules [31]. The diversity analysis of 1500 randomly sampled compounds of each library, using t-distributed stochastic neighbor embedding (tSNE) employing Molecular ACCess System (MACCS) keys [32], showed that the compounds from the tetrazole and Ugi libraries display similar structural diversity to the compounds inside DrugBank. The K-means clustering showed that compounds from the CCC and DrugBank libraries distribute similarly on five out of six clusters, whereas the sixth cluster was enriched in DrugBank small molecules (Figure 2A).

A normalized principal moments ratio (NPR) analysis was conducted to assess the molecular shape distribution of compounds. The results showed that the minimum energy conformers of the compounds from the three libraries presented similar 3D shapes, predominantly rod- and disk-shaped, with only a few compounds displaying a spherical shape (Figure 2B). We also performed a principal components analysis (PCA) employing non-redundant molecular descriptors selected by their correlation (Figure S2). PCA showed that the compounds in CCC-generated libraries possess similar molecular descriptors and physicochemical properties to those of the DrugBank Database, displaying a dense accumulation of the compounds at the origin of the principal components (PCs), PC 1 and PC2. Topological polar surface area (TPSA) and logarithmic partition coefficient (LogP) were the primary descriptors, correlating positively with PC1 and PC2 and negatively with PC2, respectively (Figure 2C).

Finally, the extended Lipinski’s rule of five (Ro5) analysis showed that most newly designed compounds comply with the physicochemical properties required for oral use [33]. Interestingly, the CCC-generated libraries displayed a more homogeneous distribution inside the extended Ro5 than the group of compounds in DrugBank (Figure 2D).

### 2.4. Virtual Screening

**Figure 3:**
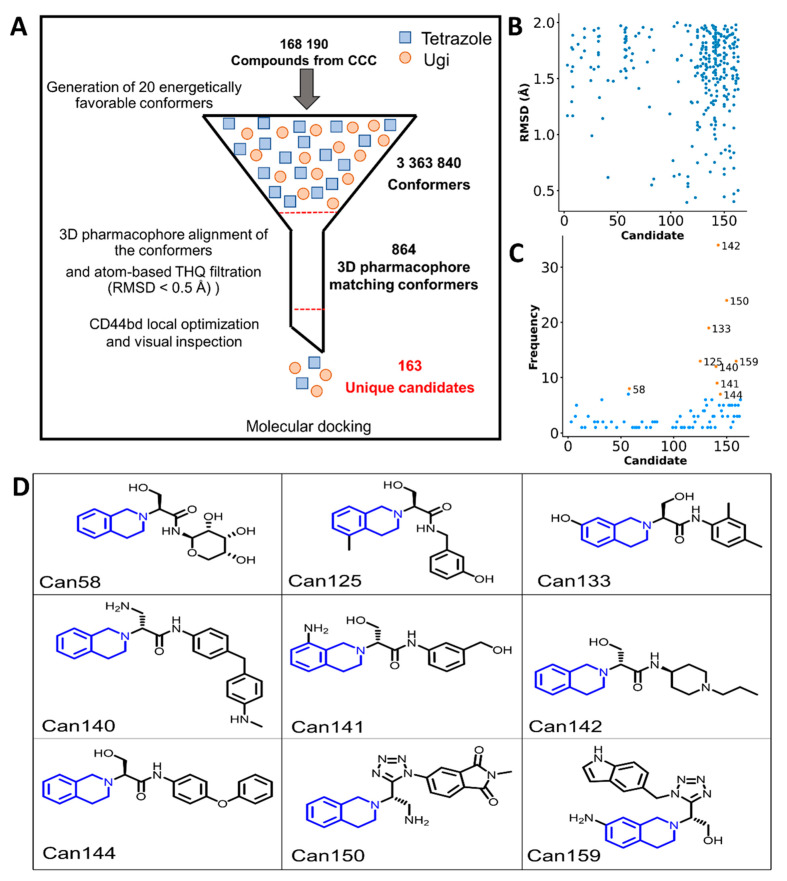
Virtual screening of THQ-containing molecules as potential CD44 antagonists. (**A**) The compounds within the CCC-generated libraries were sequentially filtered using the depicted strategy. (**B**) RMSD analysis comparing the THQ position in crystals vs. the poses obtained by docking of 163 unique compounds to human or mouse CD44HAbd. (**C**) Analysis of the frequency of poses with RMSD < 2 Å allowed the selection of nine candidates (orange dots). (**D**) Structure of the nine candidates selected by virtual screening with the THQ motif highlighted in blue.

To identify new compounds with the theoretical ability to bind CD44, we generated expanded libraries containing 20 energetically favorable conformers for each compound within the CCC-generated libraries, retrieving 3,363,840 conformers. The expanded libraries were screened by alignment to the pharmacophore, followed by local optimization in CD44HAbd and visual inspection. We identified 864 conformers from 163 unique compounds that matched the selection criteria (Figure 3A). Only those molecules were employed for the subsequent experiments.

The docking protocol employed for filtering was validated by docking the 21 THQ-containing molecules co-crystallized with mCD44HAbd into protein 5BZM. We observed that the crystal pose was more frequently reproduced in compounds with smaller substituents on the THQ motif (Figure S3A) Thus, additional analysis of candidates considered only the position of the THQ atoms.

The 163 candidates were docked against hCD44HAbd and mCD44HAbd for comparison. For each candidate/receptor pair, the docking scores of 25 poses were analyzed (Figure S4). The docking poses were compared to the coordinates of THQ crystalized on mCD44HAbd since none of the available human crystal structures contained THQ-derived compounds. We focused on the compounds showing docking poses matching the crystallized THQ atoms with RMSD < 2 Å (Figure 3B). We selected the compounds with the highest frequency of matching poses, ranging from 8/50 to 34/50 (Figure 3C). For the nine selected molecules (Figure 3D and Table S2), we assessed the THQ-binding site selectivity by docking the compounds into four/five additional pockets using three relevant forms of the receptor: apo-hCD44HAbd, HA-bound mCD44HAbd, and THQ-containing molecule mCD44HAbd. With the exception of the candidate (Can) 142, all molecules were predicted to bind the region of interest with better or similar affinity than other pockets (Figure S5). We then performed pose clustering analysis (Figure S6) to identify the best pose for molecular dynamics (MD) simulations.

### 2.5. Molecular Dynamics and Free Energy Calculation

**Figure 4:**
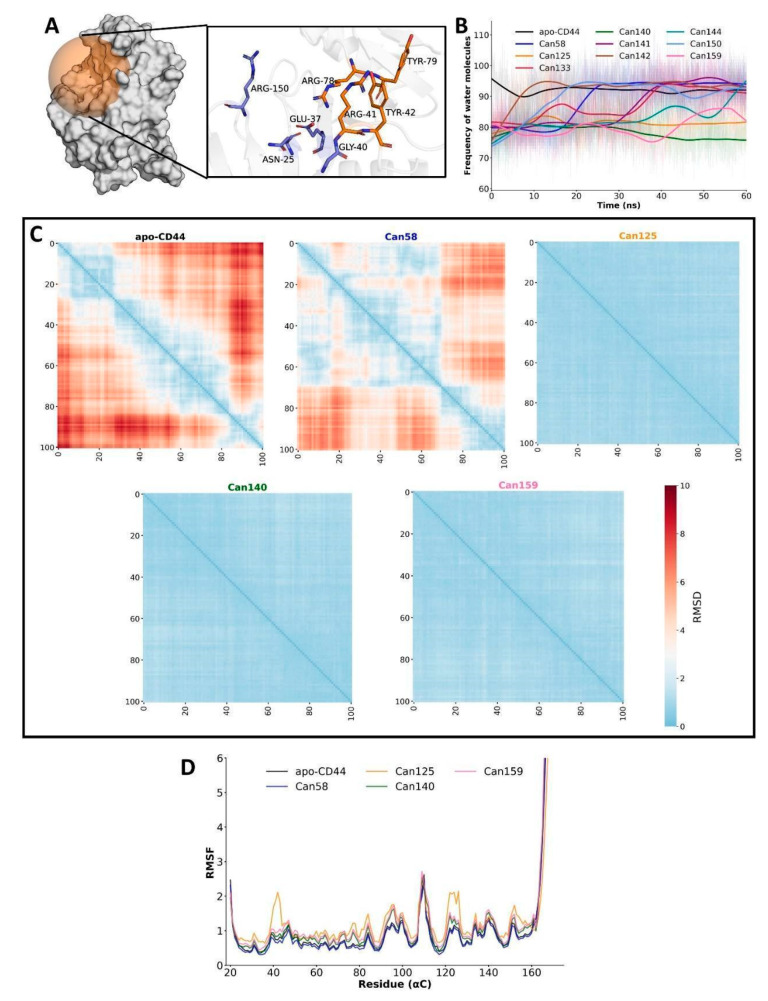
Molecular dynamics (MD) analysis identified Can 125 as a potential CD44 antagonist. (**A**) Spatial representation of the analyzed pocket in hCDHAbd. Inset shows the lateral chains of residues reported as essential for HA-binding (orange sticks) or those that mediate the interaction with the THQ-containing compounds employed for pharmacophore modeling (blue sticks). (**B**) Water molecules displacement analysis for the nine candidates shown in Figure 3D. (**C**) Pairwise backbone RMSD matrix along 100 ns of MD simulation from systems including candidates with stable binding to hCD44HAbd. The unliganded protein (apo-CD44) and the system with Can58 are included for comparison. (**D**) Alpha-carbon RMSF analysis for the systems presented in **C**.

We performed solvent explicit MD simulations to characterize the binding of the selected candidates to hCDHA44. The apo-hCD44HAbd was included as a control. Our analysis focused on the THQ binding site reported for mCD44HAbd (Figure 4A). By quantifying the water molecules displacement in the selected region, we identified that candidate Can58, Can133, Can141, Can142, and Can150 left the cavity during the simulation. Furthermore, Can58, Can142, and Can150 moved from the binding site at the early steps of the MD simulation. On the other hand, three compounds, (Can125, Can140, and Can159) remained on the binding site during the 100 ns of simulation and maintained a constant number of local water molecules (Figure 4B). Interestingly, the global backbone RMSD analysis of the systems with those three candidates showed a different profile than the one generated by apo-CD44HAbd (Figure 4C) or by systems with other candidates (Figure S7). Additionally, a shift on some residues was observed on the root mean square fluctuation (RMSF) profile generated for Can125 (Figure 4D).

**Figure 5:**
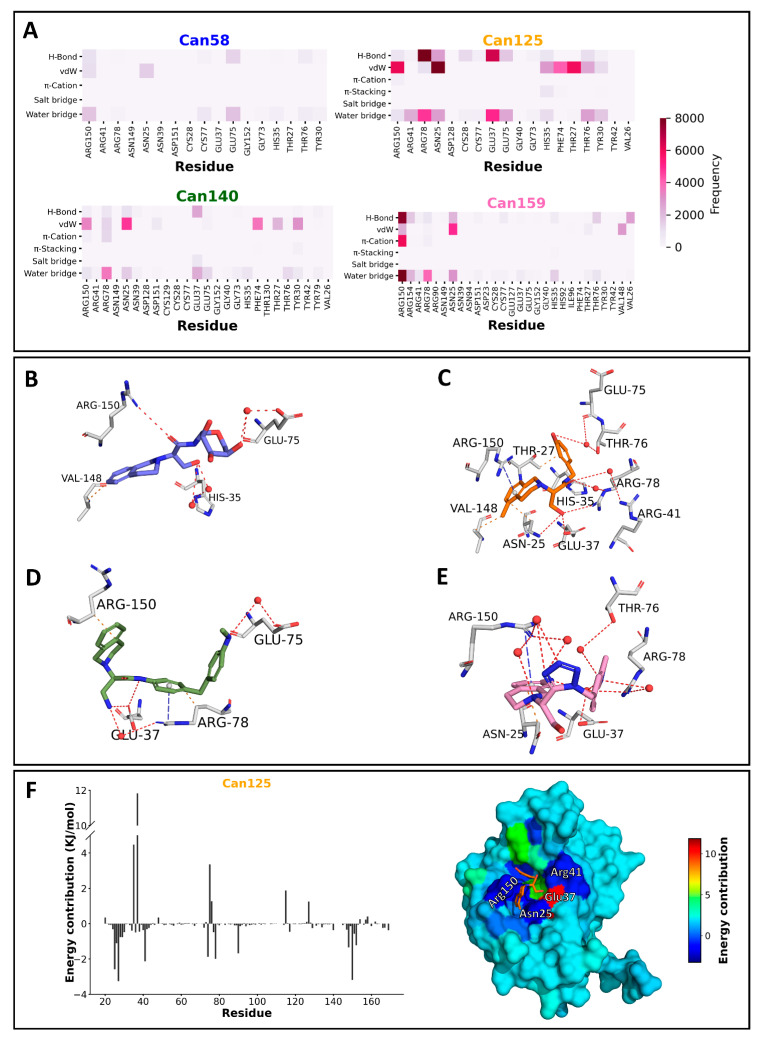
Identification and importance of residues mediating compound binding. (**A**) Types of interactions that support stable candidate/hCD44HAbd interactions and their frequency. Note that Can58, presented here for comparison, left the binding site during the simulation. (**B**–**E**) The 3D molecular interactions of a representative frame from MD simulation of the complexes between Can58 (**B**), Can125 (**C**), Can140 (**D**), and Can159 (**E**) with hCD44HAbd (gray sticks). (**F**) Per-residue energy decomposition for Can125. Most positive values correspond to His35 and Glu37, and most negative values to Asn25, Thr27, Arg41, and Arg150.

The frequencies of molecular interactions generated by Can125, Can140, and Can159 were studied during the whole simulation; Can58 was considered in the analysis for comparison (Figure S8). As expected from the water analysis, Can58 showed a low frequency of interactions in the THQ binding site, which included water bridges and hydrogen bonds with Arg150 and Glu75 (Figure 5A,B). Of the candidates studied by MD, Can125 formed the highest number of interactions, the most frequent were hydrogen bonds with Arg41 and Glu37, Van der Waals interactions with Arg150, Arg78, and Thr27, and water bridges with Arg78 and Glu37 (Figure 5A,C). Can140 showed Van der Waals interactions with residues Arg150, Asn25, and Phe74 predominantly, but it was also able to form water bridges with Arg78 and Glu37 (Figure 5A,D). Can159 showed a high frequency of π-cation, and hydrogen bonds interactions with Arg150, and water bridges with Arg78, and Arg150. Moreover, the Can159 also displayed the frequent formation of Van der Waals interactions with Asn25 (Figure 5A,E).

Calculation of the binding free energy (Table 1) showed that Can140 has lower binding energy than Can125 and Can159, with the electrostatic energy as the component that contributes most to these differences. Nevertheless, Can140 showed large energy fluctuations during the simulations (Figure S9), suggesting a possible rearrangement of the binding pose during the experiment. In contrast, Can125 and Can159 showed stable energetic profiles (Figure S9). Per-residue energetic decomposition (Figure 5F and Figure S10) revealed that Can125 binding to hCD44HAbd is supported by energetically favorable interactions with residues essential for HA-binding (Arg41 and Arg78) or residues selected in the pharmacophore modeling (Arg150). Together, these observations suggest that Can125 is the most promising compound for biological evaluation.

## 3. Discussion

Due to the essential physiological and pathological roles of CD44, several crystal structures of its HAbd have been solved, either in the unligated form (apo) or in complex with HA. Our structural analysis of the crystals available at PDB corroborated the previously identified shifts in residues participating in HA-binding, including Arg41, Tyr42, Arg78, and Tyr79 in hCD44HAbd, and Arg45, Tyr46, Arg82, and Tyr83 in mCD44HAbd [22,23,24,34]. We found the shifts in Arg41 and Arg78 as particularly important for drug design because (i) the shift in Arg41 has been identified as a trigger for the conversion of high (active) to low (inactive) affinity conformations of CD44HAbd [22] and (ii) both residues are close to a pocket that binds to small molecules with the THQ motif and suffer conformation changes induced by ligand binding.

The THQ-binding pocket has been employed for the development of molecules that display a similar affinity for human or mouse CD44HAbd (ranging from 0.4 µM to 6.9 µM for hCD44HAbd, and 0.5 µM to 11.2 µM mCD44HAbd) [24]. Thus, we used 21 murine crystals containing THQ-based molecules for developing a pharmacophore that contained the key interactions mediating the binding of those compounds to mCD44HAbd and hypothesized that the model could be used in the identification of new antagonists for the human version of the receptor.

By using MCR-based CCC, we also generated libraries of easily synthesizable compounds that contain the THQ substructure. MCR are one-pot reactions in which two or more starting materials are used simultaneously; thus, most of the atoms from the initial building blocks are incorporated into the final product of the reaction [35,36]. The Ugi four-component tetrazole synthesis [28,29] and Ugi three-component reaction [30] are well described, easy to perform, and have been suggested as synthesis methods for diverse drug-like molecules [36]. Additionally, we selected building blocks that are commercially available at low-cost, which will allow compound synthesis and activity evaluation in further studies. Chemoinformatic characterization of the generated databases showed that the compounds are highly diverse but contain similar structural and physicochemical characteristics to those of marketable molecules. The DrugBank Database drugs frequently adopt rod- and disc-shapes [37] and comply with Lipinski’s Ro5 [33,38]. The compounds within our CCC-generated libraries predominantly displayed these shapes, due to the high predominance of non-cyclic molecules [37,39], and have drug-like physicochemical characteristics. Molecules with the THQ substructure have been identified as nicotinic [40] or muscarinic [41] receptor antagonists, in addition to inhibitors of the angiotensin-converting enzyme [42]. Thus, the databases reported here may be useful starting points for identifying new compounds with those activities.

A robust exploration of the conformational space allowed the selection of 163 unique candidates that matched with the pharmacophoric model. To overcome the lack of structural information regarding the binding mode of THQ-containing molecules to hCD44HAbd, we investigated the binding mode of the candidates in hCD44HAbd and mCD44HAbd by a docking protocol that was able to reproduce the crystallographic binding mode of most co-crystallized molecules with an all-atoms RMSD threshold < 3Å. We identified nine candidates that reproduced the THQ crystallographic pose with an RMSD < 2Å and high frequency. A similar strategy, using a THQ-based pharmacophore for screening identified potential anticonvulsant compounds [43].

MD in explicit solvent further characterized the binding capability of the best nine candidates from virtual screening. We applied this method considering that the effects of solvation play a key role in forming molecular interactions in ligand–protein complexes. Thus, simulations employing explicit solvent allow the study of the most realistic and detailed level of physical chemistry of solvation [44]. We found that only three candidates (Can125, Can140, and Can159) remained bound to the THQ binding site during the entire MD simulation. In contrast, the Can142 left the THQ binding site at an early stage of the MD simulations; this observation might correlate with the fact that this candidate also showed higher docking scores for other pockets in CD44HAbd than the THQ-binding pocket (Figure S5).

Moreover, the candidates that remained bound during all simulations induced drastic decreases in the RMSD values of the hCD44HAbd backbone compared with those of the apo structure. The ligand-induced transition to a less flexible conformation of the protein can modulate its activation and improve both the compound’s affinity and residence time [45,46]. Hence, a reduction in the target’s conformational dynamics is a desirable characteristic of a drug-like molecule.

Although Can125, Can140, and Can159 displayed molecular interactions with residues involved in the HA-binding, including some reported as essential, only Can125 and Can159 reproduced the interactions predicted by the pharmacophore. Per-residue energetic decomposition corroborated that residues at the THQ-binding pocket support the binding of these two candidates to hCD44HAbd. However, only Can125 originated an RMSF profile that diverged from the unligated hCD44HAbd; specifically, it induced fluctuations in residues Arg41 and 120–126. The ligand-induced shift on Arg41 was different from the one identified on the crystal structures containing HA, which is considered essential for the transition from inactive to the active state in CD44 [22]. The changes in residues 120–126, which comprise a loop adjacent to the THQ-binding pocket, may participate in target constraint since they do not contribute to the ligand-binding energy. We hypothesize that the conformational changes induced by Can125, especially on Arg41, may impede the binding of the HA to hCD44HAbd.

On the other hand, Glu37 contributed negatively to the binding energy of Can125, which may be caused by the method employed for energy calculations. Calculations performed in implicit solvent offer a fast approach for binding energy assessment but are not yet well parameterized for complex problems that consider the presence of all solvent molecules [44]. Thus, our calculations may be underestimating the energetic contribution of water bridges and hydrogen bonds formed between the side chain of Glu37 and the methyl alcohol of Can125. Moreover, it is also possible that the proximity between opposite hydrogen bond acceptors (the carboxylate of Glu37 and the oxygen in the acetamide group of Can125) represents an unfavorable energetic contribution. This finding represents an opportunity to improve the chemical features of potential antagonists of CD44 to be proposed in future studies.

We did not assess the possible effect of the best candidates in the binding of other CD44 ligands, as aggrecan, osteopontin, collagen, or CD74 [3,47,48], given the lack of corresponding structural data. However, we found that the THQ-binding site was able to allocate the best-ranked poses of Can125 and Can159 among all CD44HAbd pockets, suggesting a better affinity for this site over other regions of CD44HAbd. Thus, we speculate that these compounds may elicit competitive inhibition only for ligands with binding sites overlapping with that of HA, such as aggrecan [49]. On the other hand, we do not have evidence to propose that binding sites outside the CD44HAbd could be affected by the candidates, although the compounds restricted the conformational dynamics of CD44HAbd.

## 4. Materials and Methods

### 4.1. Sequence and Structural Alignments

Human and murine CD44 binding-domain (hCD44HAbd and mCD44HAbd, respectively) crystal structures were retrieved from the Protein Data Bank (PDB). All structures were aligned using UCSF Chimera 1.14 [50] and the A chain of the entry 1UUH as reference. RMSD was calculated for alpha-carbons, backbone, and all atoms. Sequence alignments were performed using the pairwise2 module of Biopython 1.78 [51]. For small-molecule atom-based alignment and comparison, a python tailored-made script was created employing the Cheminformatics Toolbox RDKIT (http://www.rdkit.org, accessed date 25 February 2021) and the Pymol 2.4 API (PyMOL Molecular Graphics System, Schrödinger, LLC). The script is available at https://github.com/AngelRuizMoreno/CD44_antagonist (accessed date 25 February 2021).

### 4.2. The 3D Pharmacophore Modeling

The 3D pharmacophoric model was generated by using 21 mCD44HAbd crystal structures containing small molecules with a THQ motif (Table S1) and the Pharmit server [52]. The most relevant molecular features were chosen by visual inspection, and their 3D coordinates in the mCD44HAbd pocket were set using the threshold of RMSD < 0.5 Å among THQ atoms.

### 4.3. Combinatorial Computational Chemistry

The creation of compound libraries by the CCC approach was carried out using Reactor 20.17 from ChemAxon (http://www.chemaxon.com, accessed date 25 February 2021). The Ugi Tetrazole and Ugi 3 component reactions were selected as synthesis routes to generate compounds synthetically accessible by MCR. For the CCC experiments, libraries of building blocks were made by searching highly diverse and low-cost commercially available starting materials using the sci-finder platform (https://scifinder.cas.org). The building blocks library consisted of 32 substituted 1,2,3,4-THQ, 4 aldehydes, and 657 isocyanides.

### 4.4. Cheminformatic Analysis

The resulting compounds from CCC were stored in two different libraries according to their synthesis origin. For comparison, the DrugBank dataset was included in the cheminformatic analysis. For each library, we computed tSNE, NPR, PCA, and Ro5. The tSNE analysis was computed using the Tanimoto similarity among the MACCS keys for each molecule using RDKIT; then, the tSNE calculation was conducted using Scikit learn 0.23.1 [53], implementing two components and a perplexity value of 50. After tSNE analysis, a silhouette-based k-means clustering was performed using Scikit learn 0.23.1 [53]. Similarly, the PCA was performed using Scikit learn and employing ten selected non-redundant molecular descriptors of 30 different 2D and 3D molecular descriptors, which were computed using RDKIT. The NPR analysis was carried out by calculating the NPR1 and NPR2 descriptors for the molecules. Finally, the Ro5 analysis was performed by calculating the molecular weight, LogP, number of hydrogen bond donors and acceptors, and TPSA.

### 4.5. The 3D Pharmacophoric Matching

We generated 20 energetically favorable conformers for each compound within our working libraries by using the Mcnf module of Moloc [54]. All the conformers were aligned against the THQ-atoms and pharmacophore using the Pharmit server [52]. The best conformers were selected using an RMSD threshold of 0.5 Å against the pharmacophoric model descriptors. Finally, a local optimization was performed using the mCD44HAbd surface using Moloc [54,55], followed by visual inspection. The molecules that after local optimization kept the 3D pharmacophoric matching (RMSD < 0.5 Å) were selected for further studies.

### 4.6. Molecular Docking

According to the identity and RMSD values, we found a very high similarity between all hCD44bd. Therefore, we choose the first hCD44bd crystal reported (1UUH). Regarding the mCD44HAbd our data also indicated a very high similarity among all available structures. Nonetheless, for docking experiments, we focused on the mCD44HAbd containing THQ-molecules, and we choose the crystal 5BZM, which contains a THQ-molecule displaying all molecular features according to our pharmacophoric model.

For the validation of the molecular docking protocol, the crystal structure of mCD44HAbd (5BZM) was used as the receptor and 21 co-crystallized THQ-containing molecules as ligands. For virtual screening, proteins hCD44HAbd (1UUH) and mCD44HAbd (5BZM) were employed as receptors, and the 163 molecules selected by pharmacophore filtering as ligands. Secondary dockings were carried out into additional pockets identified Fpocket v3.0 [56] in ligand-free hCD44HAbd (1UUH), HA-bound mCD44HAbd (2JCR), or THQ-containing molecule mCD44HAbd (5BZM).

Proteins were prepared by removing co-crystallized waters, solvent molecules, and adding charges and hydrogens using Chimera 1.14 [50]. Ligands were prepared by adding explicit hydrogens and tautomeric states at pH 7.4. and generating 3D coordinates with Standardized 19.20.0 (http://www.chemaxon.com, accessed date 25 February 2021). For virtual screening, docking was performed into hCD44HAbd and mCD44HAbd within a sphere with 8 Å of radius and center in the Thr27 or Thr31, respectively. Secondary dockings were carried out using as reference the coordinates of each additional pocket identified. For each ligand, 50 runs of the genetic algorithm were performed for the conformational search. Each pose was evaluated employing the PLP Chemscore scoring function established in the GOLD software from the Cambridge Crystallographic Data Center (CCDC) [57]. For each compound, the best 25 poses were saved for analysis. Finally, hierarchical clustering analysis of the poses was performed using Scipy 1.5.2 [58].

### 4.7. Molecular Dynamics

The MD simulations were carried out using Gromacs 5.0.4 [59]. Selected candidates and hCD44HAbd (1UUH) were parameterized using the CGenFF and CHARMM36 force field, respectively, through the CHARMM-GUI (http://www.charmm-gui.org/, accessed date 25 February 2021) [60]. The systems were built by adding TIP3P water molecules to the ligand-hCD44HAbd complexes, neutralizing ions, and establishing periodic boundary conditions (PBC) by using the multicomponent assembler of the CHARMM-GUI. Before production, the systems were minimized and then equilibrated under an NVT assembly. During the production phase, an NPT assembly was performed at 310.15 K for 100 ns saving velocities, energy, and positions every ten ps. Water molecules displacement was computed by quantifying the number of water molecules within a 6 Å radius sphere covering the THQ-binding pocket, every frame of the simulation. Analysis of ligand-target interactions was carried out by a python tailored-made script (https://github.com/AngelRuizMoreno/CD44_antagonist, accessed date 25 February 2021) implementing MDAnalysis [61] and PLIP [62].

### 4.8. Interactions Free Energy Calculations

Full-length candidate-hCD44HAbd trajectories were employed for free energy calculations using the molecular mechanics energies combined with the Poisson–Boltzmann surface area continuum solvation (MM/PBSA) [63] by g_mmpbsa v1.6 package [64]. Computation of the potential energy in vacuum, polar solvation energy, and non-polar solvation energy were performed to calculate the average binding energy. Per-residue energetic decomposing and maps were created to show the contribution of each residue to the binding energy.

## 5. Conclusions

Our experiments demonstrate that the compounds Can125 [3-hydroxy-N-(3-hydroxybenzyl)-2-(5-methyl-3,4-dihydroisoquinolin-2(1H)-yl)propenamide] and Can159 [2-(1-((1H-indol-5-yl)methyl)-1H-tetrazol-5-yl)-2-(7-amino-3,4-dihydroisoquinolin-2(1H)-yl)ethan-1-ol] bind with high theoretical affinity to the murine and human structures of CD44HAbd and stabilize the conformational dynamics of the protein. Therefore, those compounds may elicit a blocking effect on HA-binding.

## Figures and Tables

**Table 1:** Average free energy calculated from MD simulations (mean ± standard deviation).

Compound	Van der Waals Energy	Electrostatic Energy	Polar Solvation Energy	SASA Energy	Binding Energy
Can58	−33.871 ± 35.190	−18.447 ± 29.906	47.592 ± 66.887	−4.667 ± 4.853	−9.393 ± 41.581
Can125	−116.512 ± 15.988	−52.356 ± 23.296	123.555 ± 27.579	−13.962 ± 1.252	−59.274 ± 17.744
Can140	−96.931 ± 36.947	−128.983 ± 66.843	106.522 ± 90.455	−12.078 ± 4.022	−131.470 ± 41.310
Can159	−99.395 ± 18.056	−34.928 ± 25.315	112.733 ± 40.928	−11.318 ± 1.938	−32.908 ± 17.750

## Data Availability

All data, including the in silico libraries, are available from the authors upon request.

## Supplementary Materials

The following are available online: Table S1: mCD44HAbd crystal structures employed for 3D pharmacophoric modeling, Figure S1: Sequence and structural analysis of CD44HAbd crystals, Figure S2: Correlation matrix of the molecular descriptors from CCC-libraries, Figure S3: Validation of the docking protocol, Figure S4: Ligand efficiency from docking scores for the 163 unique candidates, Table S2: SMILES codes and formal names of the nine selected candidates, Figure S5: Comparison of theoretical binding of the best nine candidates among multiple pockets within CD44HAbd, Figure S6: Docking pose clustering for the best nine candidates, Figure S7: Pairwise backbone RMSD matrix along 100 ns of MD simulation and alpha-carbon RMSF analysis, Figure S8. Frequency analysis of molecular interactions, Figure S9: Energy calculations generated from MD simulations, Figure S10: Per-residue energy decomposition for the MD-simulated binding of candidates.
