# Supplementary material for: In Silico Design and Selection of New Tetrahydroisoquinoline-Based CD44 Antagonist Candidates
Source: Molecules. 2021 Mar 26;26(7):1877. doi: 10.3390/molecules26071877 (PMC8037692; doi:10.3390/molecules26071877)
Supplement: Supplementary file 1 [file molecules-26-01877-s001.pdf]

In Silico Design and Selection of New Tetrahydroisoquinoline-Based CD44 Antagonist Candidates

Angel J. Ruiz-Moreno [1,2,3,4], Atilio Reyes-Romero [4], Alexander Dömling [4,*], and Marco A. Velasco-Velázquez [1,2,*]

[1] Departamento de Farmacología, Facultad de Medicina, Universidad Nacional Autónoma de Mexico (UNAM), 04510 Ciudad de Mexico, Mexico
[2] Unidad Periférica de Investigación en Biomedicina Translacional, Facultad de Medicina, Universidad Nacional Autónoma de México (UNAM), Félix Cuevas 540, 03229 Ciudad de Mexico, Mexico
[3] Doctorado en Ciencias Biomédicas, Universidad Nacional Autónoma de México (UNAM), Ciudad Universitaria, 04510 Ciudad de Mexico, Mexico
[4] Drug Design Group, Department of Pharmacy, University of Groningen, 9700 AD Groningen, The Netherlands
* Correspondence: a.s.s.domling@rug.nl (A.D.); marcovelasco@unam.mx (M.A.V.-V.);
Tel.: +31-50-363-330 (A.D.); +52-55-5623-2282 (M.A.V.-V.)

Supplemental Information

Supplemental Table S1. mCD44HAbd crystal structures employed for 3D pharmacophore modeling

PDB	Resolution	Year	Reference
**4MRE**	1.58 Å	2014	[24]
**4MRF**	1.55 Å	2014	[24]
**4MRG**	1.69 Å	2014	[24]
**4NP2**	1.75 Å	2014	[24]
**4NP3**	1.61 Å	2014	[24]
**5BZC**	1.95 Å	2016	To be published
**5BZE**	1.31 Å	2016	To be published
**5BZF**	2.77 Å	2016	To be published
**5BZG**	2.19 Å	2016	To be published
**5BZH**	1.95 Å	2016	To be published
**5BZI**	1.32 Å	2016	To be published
**5BZG**	1.40 Å	2016	To be published
**5BZK**	1.40 Å	2016	To be published
**5BZL**	1.23 Å	2016	To be published
**5BZM**	1.25 Å	2016	To be published
**5BZN**	1.23 Å	2016	To be published
**5BZO**	1.22 Å	2016	To be published
**5BZP**	1.23 Å	2016	To be published
**5BZQ**	1.20 Å	2016	To be published
**5BZR**	1.15 Å	2016	To be published
**5BZS**	1.50 Å	2016	To be published
**5BZT**	1.25 Å	2016	To be published

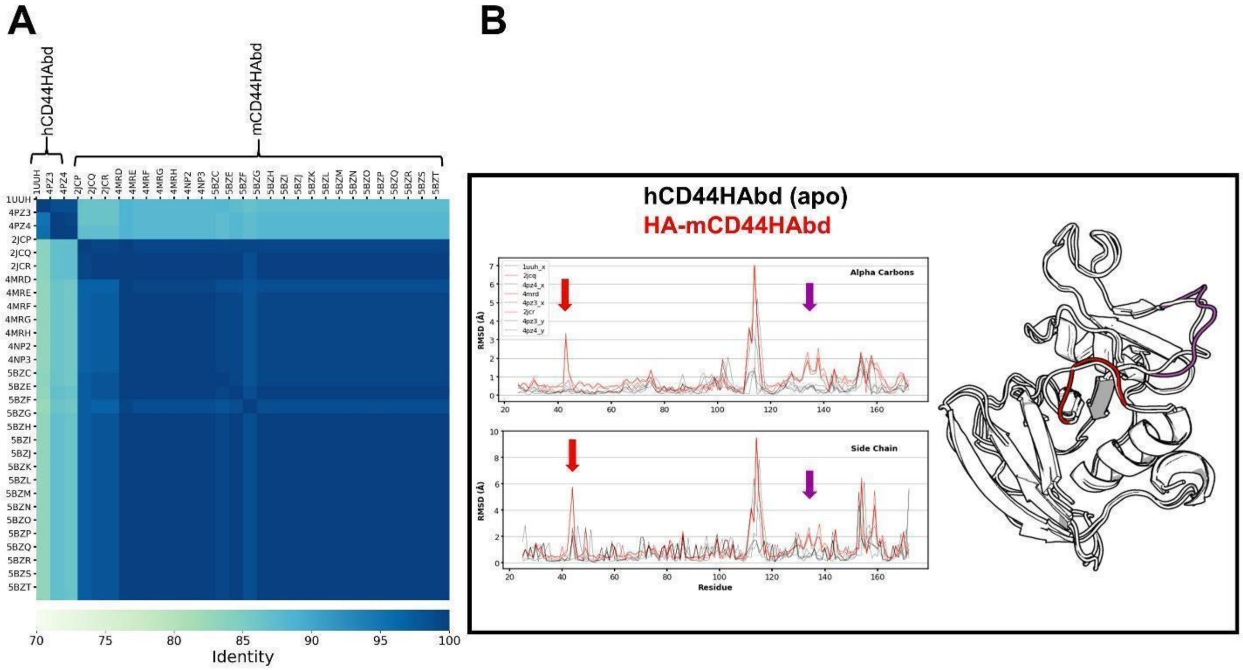

Supplemental Figure S1. A) Comparison of the primary sequences of human (h) and mouse (m) CD44 HA-binding domain (CD44HAbd) available at PDB. **B**) RMSD calculated for selected ligand-free hCD44HAbd (apo) vs. HA-bound mCD44HAbd crystals (all structures co-crystallized with HA are murine). Two regions with significant structural changes associated with HA binding are indicated with arrows in graphs and colored in ribbons structure.

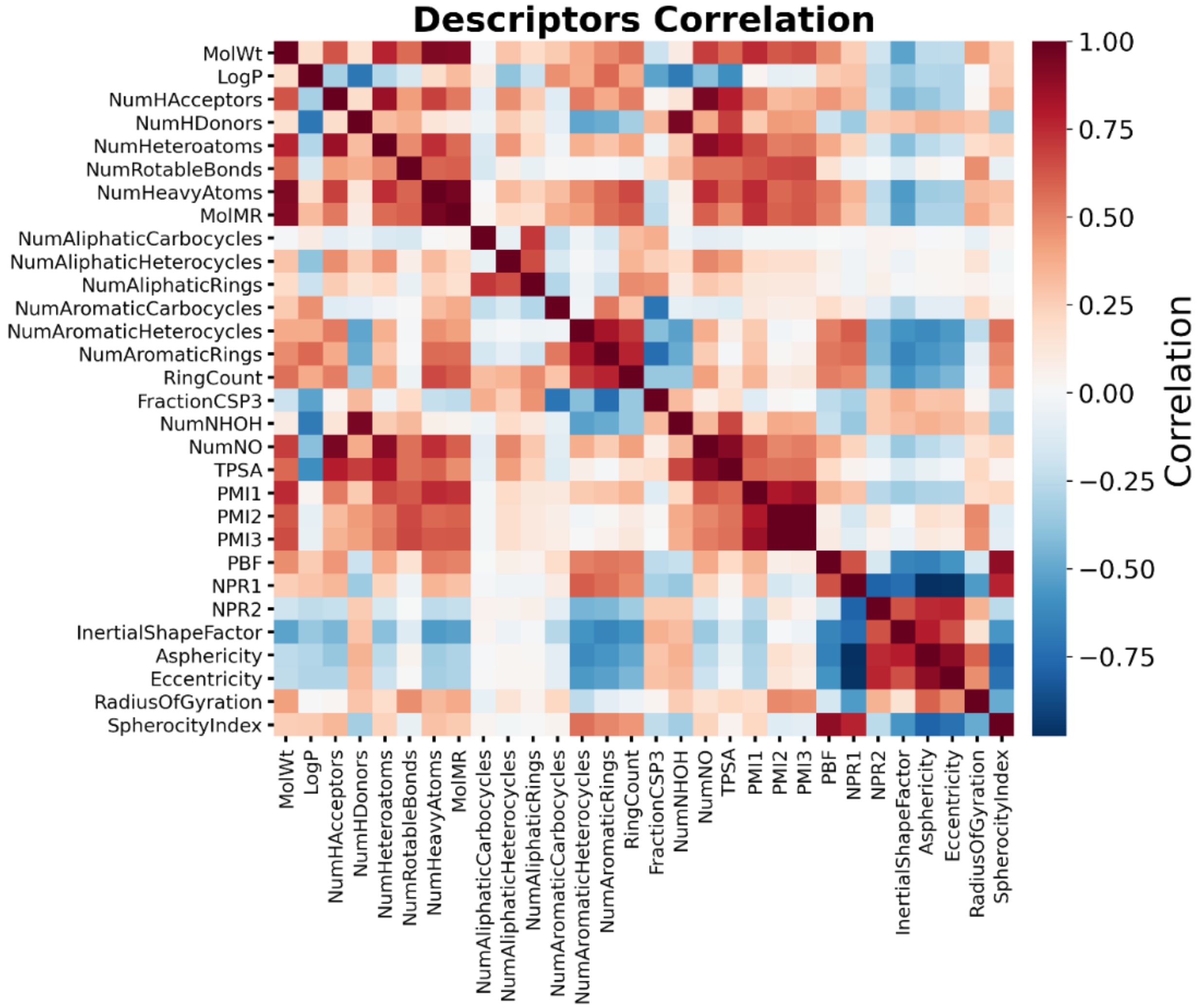

Supplemental Figure S2. Correlation matrix of the molecular descriptors from the 168,190 compounds contained in our CCC-generated libraries. The matrix was employed for the selection of the non-redundant descriptors included in PCA presented in Figure 2.

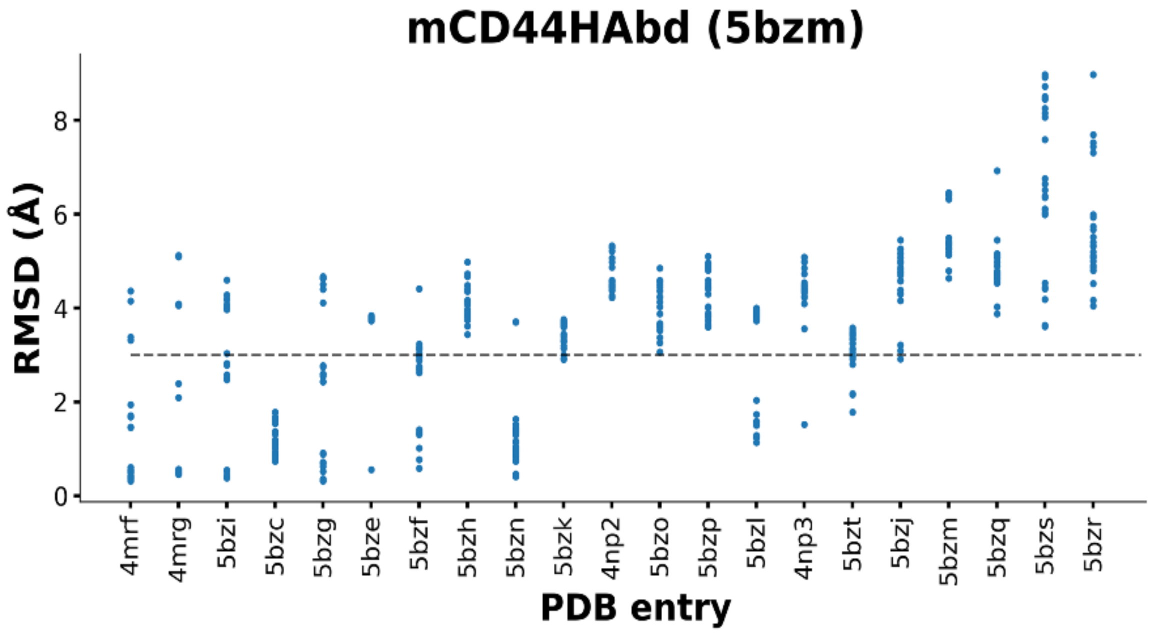

Supplemental Figure S3. Validation of the docking protocol employed for candidate selection. RMSD of docking poses vs. crystal pose of 21 THQ-containing molecules. Molecules are identified by the PDB code in which they appear as ligands and are ordered from low (left) to high (right) molecular weight. Dashed line indicates the threshold considered for analysis.

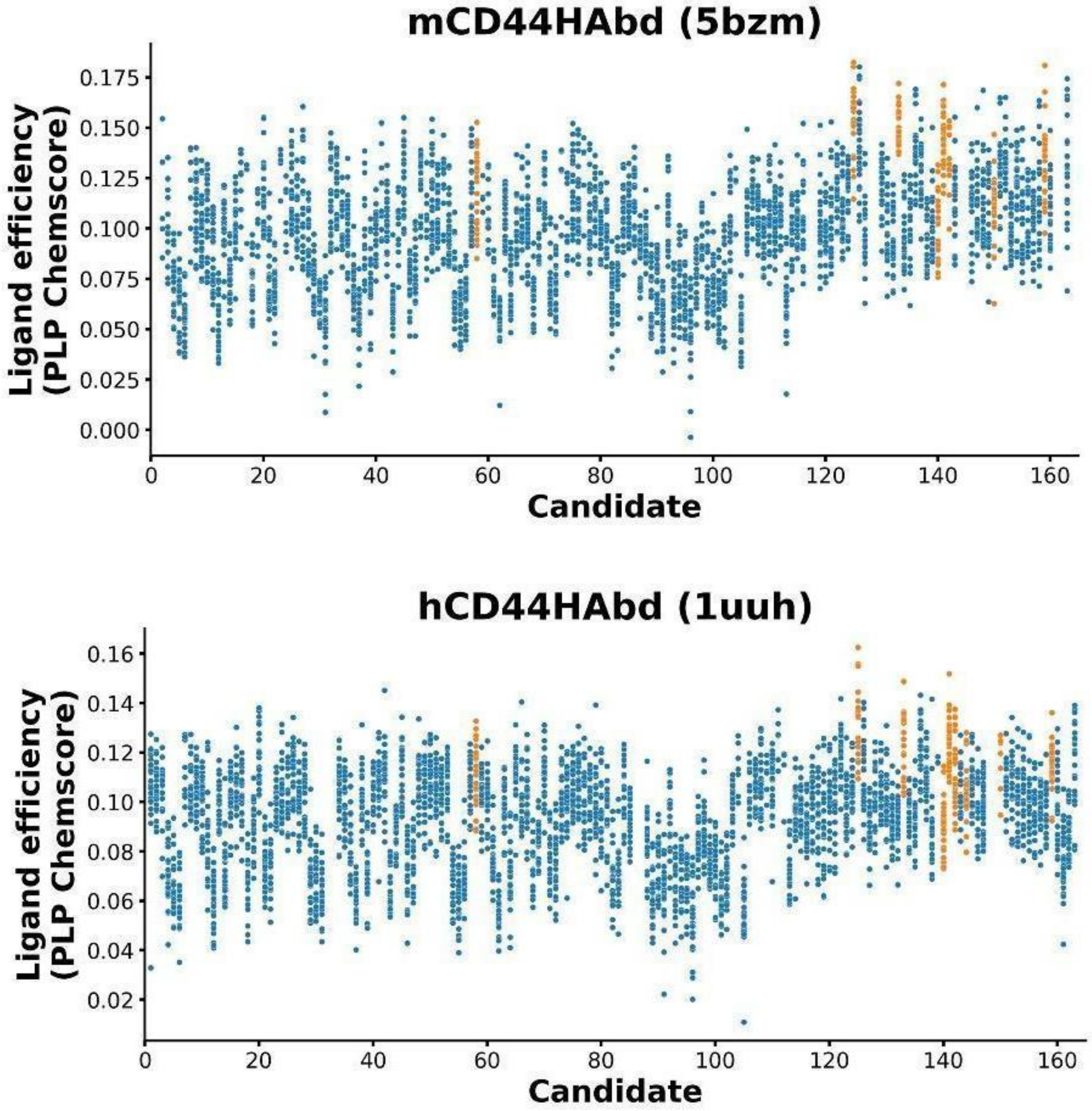

Supplemental Figure S4. Ligand efficiency, calculated from docking scores, for the 163 unique candidates matching our 3D pharmacophore. The 25 best poses on mCD44 (**A**) and hCD44 (**B**) were analyzed. Values of the candidates selected for further analysis (see Figure 3) are labeled in orange.

Supplemental Table S2. SMILES codes and formal names of the nine candidates presented in Figure 3D.

Code	SMILES	Name
**Can58**	OCC(C(N[C@@H](C1O)OCC(C1O)O)=O)N(C2)CCC3=C2C=CC=C3	2-(3,4-dihydroisoquinolin-2(1H)-yl)-3-hydroxy-N-((2R)-3,4,5-trihydroxytetrahydro-2H-pyran-2-yl)propanamide
**Can125**	CC1=CC=CC2=C1CCN(C2)C(C(NCC3=CC=CC(O)=C3)=O)CO	3-hydroxy-N-(3-hydroxybenzyl)-2-(5-methyl-3,4-dihydroisoquinolin-2(1H)-yl)propanamide
**Can133**	CC1=CC=C(C(C)=C1)NC(C(CO)N2CCC3=CC=C(C=C3C2)O)=O	N-(2,4-dimethylphenyl)-3-hydroxy-2-(7-hydroxy-3,4-dihydroisoquinolin-2(1H)-yl)propanamide
**Can140**	CNC1=CC=C(C=C1)CC2=CC=C(C=C2)NC(C(CN)N3CCC4=CC=CC=C4C3)=O	3-amino-2-(3,4-dihydroisoquinolin-2(1H)-yl)-N-(4-(4-(methylamino)benzyl)phenyl)propanamide
**Can141**	OCC1=CC(NC(C(N2CCC(C=CC=C3N)=C3C2)CO)=O)=CC=C1	2-(8-amino-3,4-dihydroisoquinolin-2(1H)-yl)-3-hydroxy-N-(3-(hydroxymethyl)phenyl)propanamide
**Can142**	CCCN1CCC(CC1)NC(C(CO)N2CCC3=CC=CC=C3C2)=O	2-(3,4-dihydroisoquinolin-2(1H)-yl)-3-hydroxy-N-(1-propylpiperidin-4-yl)propanamide
**Can144**	O=C(NC1=CC=C(C=C1)OC2=CC=CC=C2)C(CO)N3CCC4=CC=CC=C4C3	2-(3,4-dihydroisoquinolin-2(1H)-yl)-3-hydroxy-N-(4-phenoxyphenyl)propanamide
**Can150**	NCC(C1=NN=NN1C(C=C2C3=O)=CC=C2C(N3C)=O)N4CC5=CC=CC=C5CC4	5-(5-(2-amino-1-(3,4-dihydroisoquinolin-2(1H)-yl)ethyl)-1H-tetrazol-1-yl)-2-methylisoindoline-1,3-dione
**Can159**	NC1=CC=C2C(CN(CC2)C(C3=NN=NN3CC4=CC=C5NC=CC5=C4)CO)=C1	2-(1-((1H-indol-5-yl)methyl)-1H-tetrazol-5-yl)-2-(7-amino-3,4-dihydroisoquinolin-2(1H)-yl)ethan-1-ol

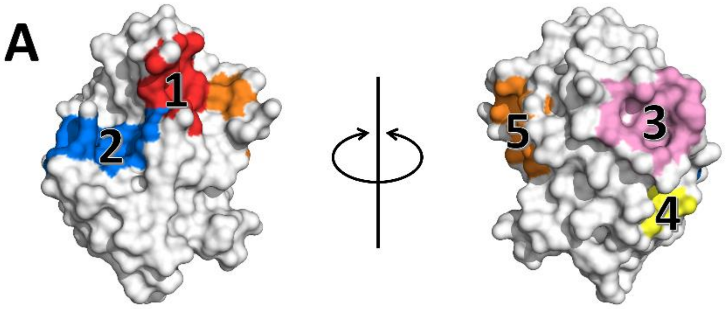

1uuh

Candidate	Score in pocket 2 (pocket rank)	Highest score in another pocket
Can58	60.25 (1)	49.64 [pocket 4]
Can125	52.75 (2)	57.34 [pocket 5]
Can133	56.18 (1)	54.82 [pocket 1]
Can140	59.02 (2)	65.81 [pocket 3]
Can141	53.66 (3)	58.85 [pocket 5]
Can142	47.61 (3)	59.20 [pocket 3]
Can144	57.10 (2)	66.13 [pocket 3]
Can150	58.26 (1)	55.86 [pocket 4]
Can159	66.75 (1)	56.95 [pocket 3]

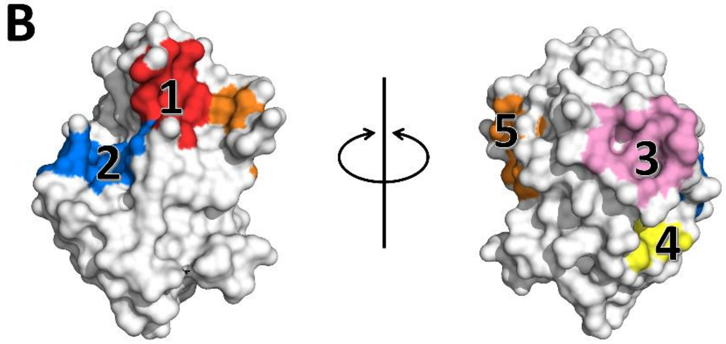

2jcr

Candidate	Score in pocket 2 (pocket rank)	Highest score in another pocket
Can58	65.44 (1)	50.98 [pocket 5]
Can125	56.24 (2)	57.91 [pocket 3]
Can133	52.75 (1)	52.17 [pocket 3]
Can140	60.35 (2)	68.30 [pocket 3]
Can141	56.25 (2)	58.91 [pocket 5]
Can142	48.72 (3)	60.13 [pocket 3]
Can144	56.58 (2)	61.74 [pocket 3]
Can150	59.24 (1)	54.63 [pocket 3]
Can159	56.63 (2)	57.88 [pocket 3]

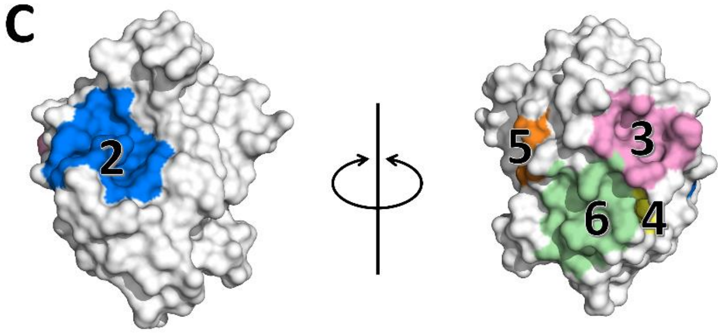

5bzm

Candidate	Score in pocket 2 (pocket rank)	Highest score in another pocket
Can58	59.03 (1)	53.92 [pocket 5]
Can125	62.72 (1)	58.92 [pocket 6]
Can133	56.41 (1)	52.81 [pocket 4]
Can140	65.85 (1)	62.71 [pocket 3]
Can141	60.56 (1)	57.47 [pocket 3]
Can142	52.33 (2)	59.67 [pocket 3]
Can144	63.00 (2)	65.01 [pocket 3]
Can150	62.37 (1)	52.67 [pocket 3]
Can159	65.52 (1)	58.72 [pocket 3]

Supplemental Figure S5. Druggable pockets in apo-hCD44HAbd (**A**), HA-mCD44HAbd (**B**), and mCD44HAbd bound to a THQ-containing ligand (**C**), as predicted by Fpocket. The THQ-binding site corresponds to pocket 2 (blue). The comparison of binding scores between pockets (tables at the right part of figures) allowed assessment of the candidates' selectivity for the region of interest.

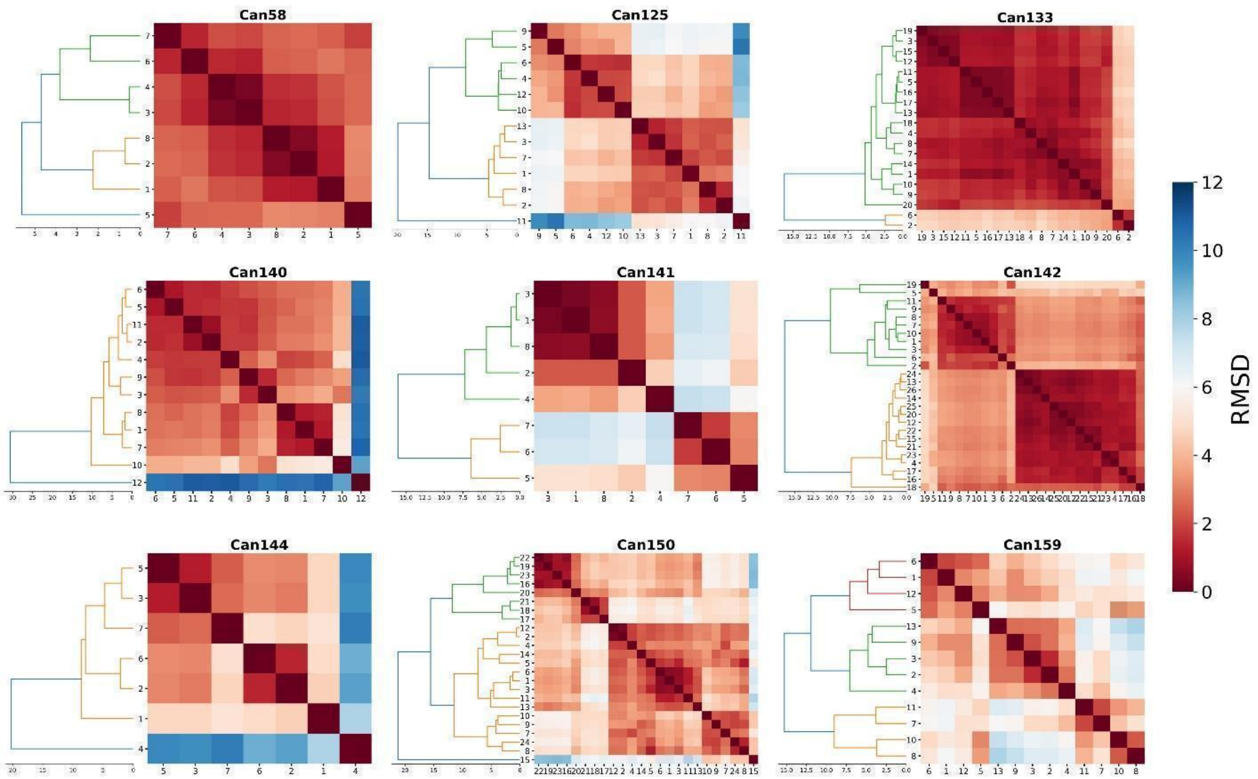

Supplemental Figure S6. Docking pose clustering for the nine candidates with the higher frequency of poses resembling the crystallographic THQ pose. Only non-redundant poses (threshold RMSD >0.2 Å) are shown. These analyses allowed the identification of the most probable starting poses for MD simulations.

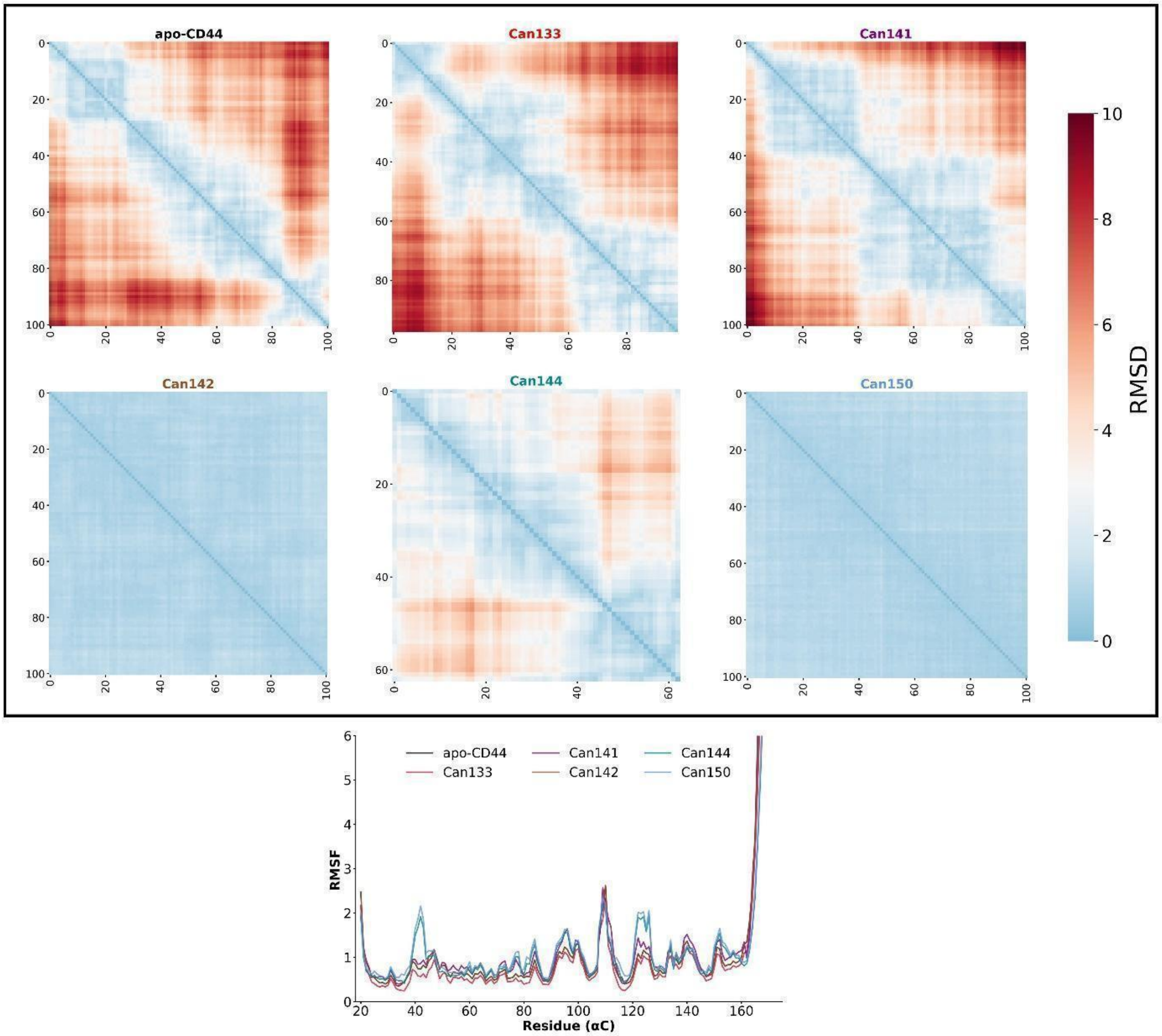

Supplemental Figure S7. Global backbone RMSD matrix along 100 ns of MD simulation, and the corresponding alpha-carbon RMSF analysis, from systems with candidates (Can) with poor binding stability. The unliganded apoprotein (apo-CD44HAbd) is included for comparison.

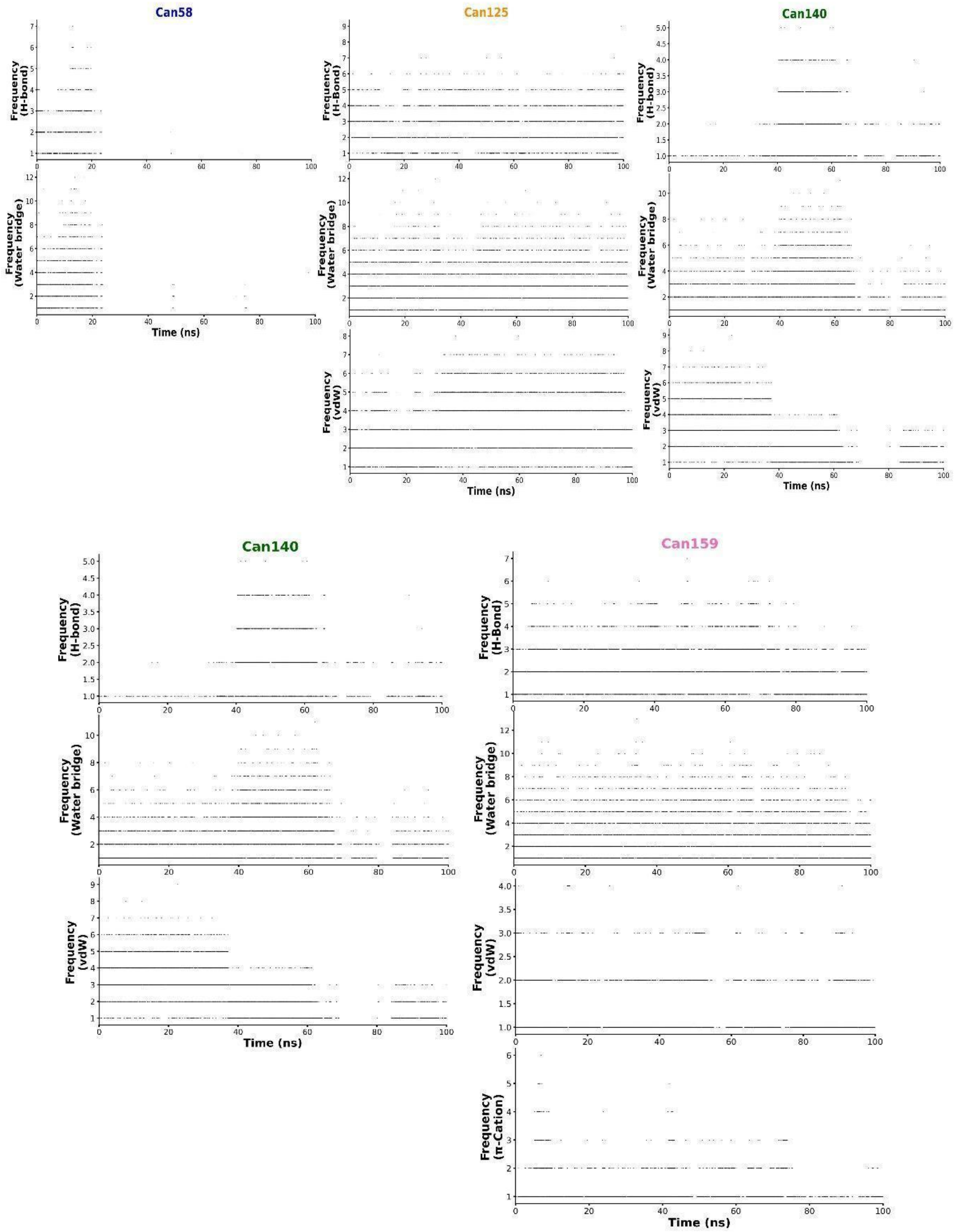

Supplemental Figure S8. Frequency analysis of interactions employed for the generation of Figure 5A. Only interactions with frequency >2,000 are shown.

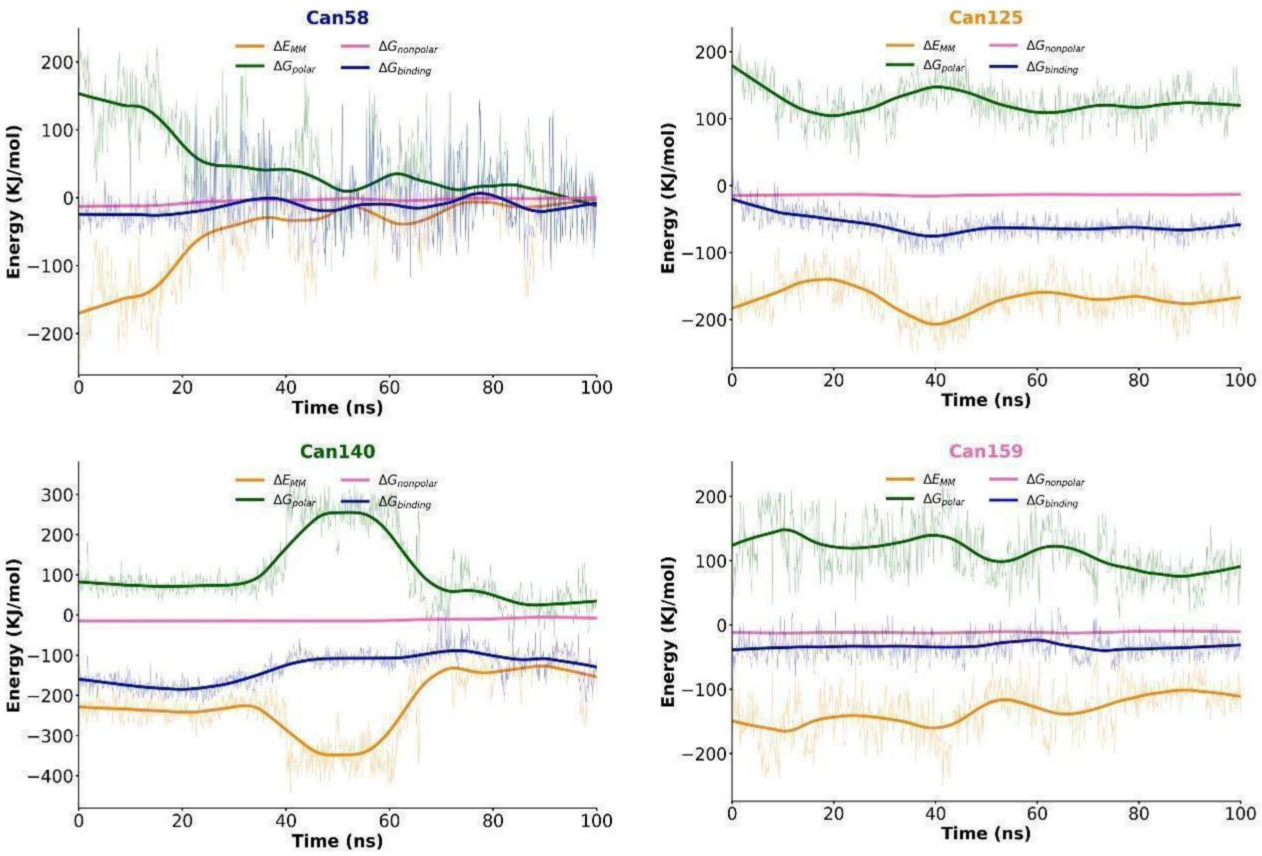

Supplemental Figure S9. Energy calculations generated from MD simulations with candidates (Can) 125, 140, and 159. Can58 was employed as a negative control since it leaves the binding site during the simulation.

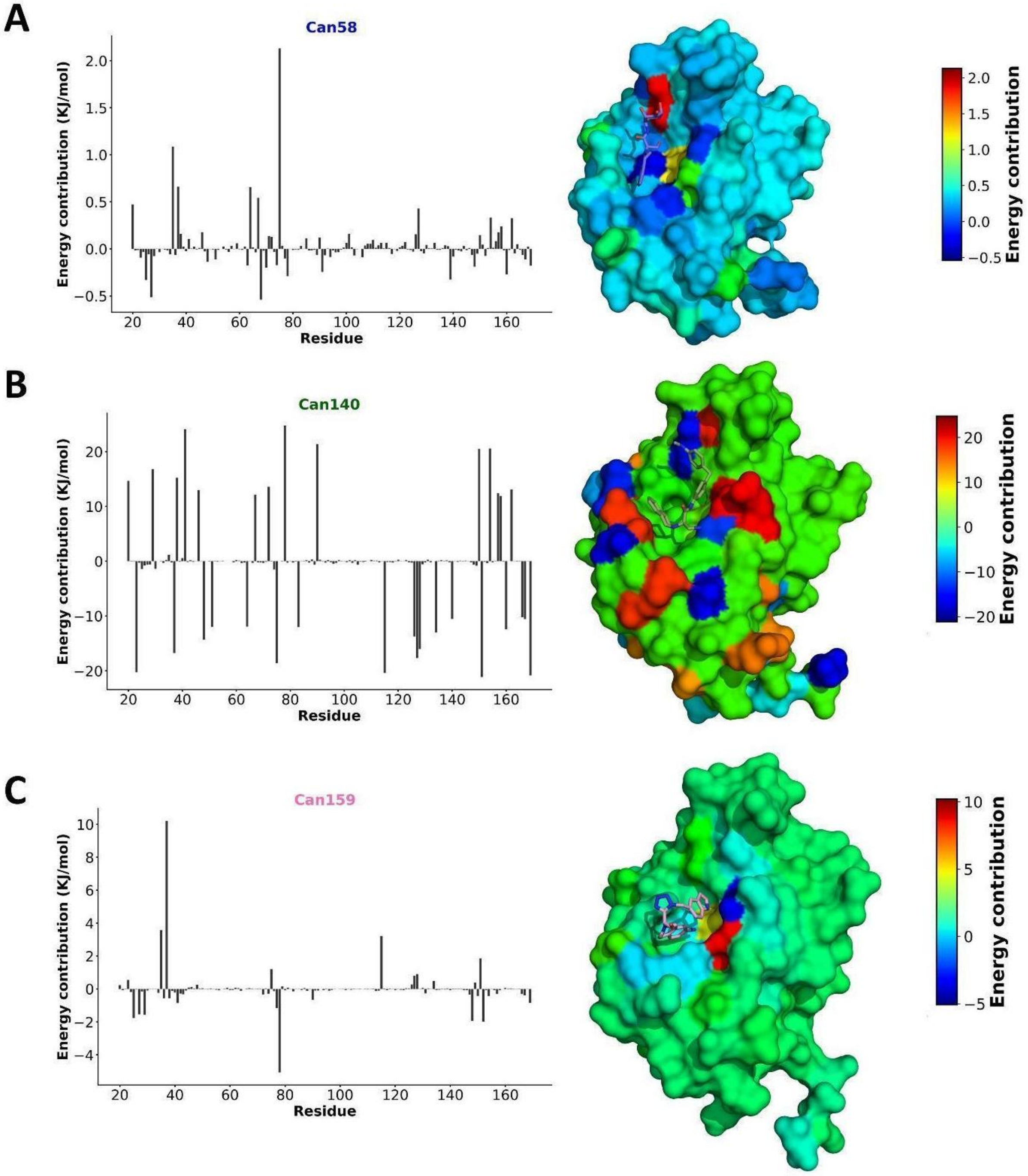

Supplemental Figure S10. Per-residue energy decomposition for the MD-simulated binding of candidates (Can) 58 (**A**), 140 (**B**), and 159 (**C**) to hCD44HAbd.
